# Up-regulation of systemic and local BDNF in non-allergic Nasal Polyps in China

**DOI:** 10.12669/pjms.40.5.7907

**Published:** 2024

**Authors:** Xiangmin Zhou, Qian Zhang, Yuzhu Wan, Li Shi

**Affiliations:** 1 Xiangmin Zhou, MM, Department of Otolaryngology, Shandong Provincial ENT Hospital, Shandong University, Shandong, China; 2 Qian Zhang, MBBS, Department of Otolaryngology, Shandong Provincial ENT Hospital, Shandong University, Shandong, China; 3 Yuzhu Wan, PhD, Department of Otolaryngology, Shandong Provincial ENT Hospital, Shandong University, Shandong, China; 4 Li Shi, PhD, Department of Otolaryngology, Shandong Provincial ENT Hospital, Shandong University, Shandong, China

**Keywords:** Nasal polyps, Non-allergic nasal polyps, Brain derived neurotrophic factor (BDNF), Cytokeratins 5 (CK5), Eosinophil cationic protein (ECP)

## Abstract

**Objective:**

Although the role of brain-derived neurotrophic factor (BDNF) in allergic rhinitis and/or nasal polyps (NPs) development has been studied, the contribution of BDNF in non-allergic NPs has not been evaluated yet. This study was to investigate the possible role of BDNF in non-allergic NPs pathogenesis.

**Methods:**

The study was carried out at The Second Hospital of Shandong University from December 2020 to November 2021. The non-allergic NPs patients (n=26) and the control group (n=22) were included. Lund-Mackay CT scores, nasal endoscopy scores, and pulmonary function testing were evaluated before surgery. Tissue and serum levels of BDNF, eosinophil cationic protein (ECP), and cytokeratins 5 (CK5) were assessed between different groups.

**Result:**

The BDNF level in serum and tissue, CK5 count, and eosinophil infiltration in tissue were higher in non-allergic NPs. The eosinophils infiltration, ECP mRNA expression level, as well as BDNF mRNA level were increased in the BDNF^high^ subgroup compared with BDNF^low^ subgroup. Significantly negative correlations between BDNF count and the situation of airway obstruction were found in non-allergic NPs.

**Conclusion:**

BDNF may have both local and systemic effects in non-allergic NPs pathogenesis. BDNF may be a possible therapeutic target or an indicator for eosinophilic NPs management.

## INTRODUCTION

Nasal polyps (NPs) are a prevalent chronic rhinologic condition that have a significant impact on patients’ quality of life and can lead to substantial medical expenses.^1^ Several comorbid conditions such as allergic rhinitis and asthma are commonly reported in patients with NPs. In this study, we define NPs without concomitant allergic diseases as non-allergic NPs.

Brain-derived neurotrophic factor (BDNF) is one of the most prominent members of the neurotrophins family. Its function not only includes synapse formation and plasticity but also extends beyond these roles to the support of aberrant innervation in many diseases associated with inflammation.^2^ Both clinical and experimental research indicates that neurosensory mechanisms are not only activated by allergic stimuli but also crucial in governing and modulating the allergic response through the release of proinflammatory cytokines and soluble mediators. As a member of the neurotrophic family, BDNF may regulate and influence the allergic response.^3^ Convincing evidence showed that Type-2 inflammation cytokines upregulated in allergic rhinitis and positively correlated with BDNF.^4^ Asian NPs usually show a mixed Th1/Th2/Th17 response with increased eosinophils, neutrophils, macrophages, and mast cell infiltration, while NPs in Western countries are commonly regarded as a Th2 profile dominated by eosinophilia.^5^ Eosinophils are one of the major effector cells to induce tissue damage, which can store, produce and release neurotrophins including BDNF.^6^ Some researchers have reported that eosinophils are a possible source of elevated neurotrophin levels in allergy and asthma.^7^ In allergic disease, BDNF generally are overexpressed in eosinophils, exerting an anti-apoptosis effect and promoting eosinophils survival.^8^

Eosinophil cationic protein (ECP), also known as RNase A family 3 (RNASE3), has been widely described and standardized as a marker of tissue eosinophilia and eosinophil activation.^9^ The biological properties and clinical role of ECP have been demonstrated. Jin reported that NPs patients had higher concentrations of ECP in nasal secretions than healthy subjects.^10^ The level of ECP, one of the eosinophil mediators, was elevated in the serum of patients with allergic rhinitis compared to healthy individuals.^11^ Non-allergic rhinitis and allergic rhinitis exhibited comparable levels of ECP and other inflammatory biomarkers.^12^

Recombinant Keratin 5 (KRT5), also recognized as Cytokeratins 5 (CK5), is expressed in a highly specific manner in epithelial cells where it plays a crucial role in the integrity and mechanical stability of the cells.^13^ Epithelial dysfunction in NPs exhibits some morphological changes, including squamous metaplasia, basal cell proliferation, goblet cell hyperplasia and the loss of differentiation of ciliated cells.^14^ Some researchers have demonstrated that the number of cells colocalized for C-C motif chemokine ligand 26 (CCL26) and CK5 was higher in NPs compared to healthy individuals.^15^

As the pathophysiological processes are likely to interfere with each other between allergic rhinitis and NPs, NPs were investigated in pure form (serum specific immunoglobulin E testing was performed to exclude allergic patients) in this study. The contribution of BDNF in non-allergic NPs has not been evaluated yet, we aimed to investigate the possible role of BDNF on non-allergic NPs pathogenesis, from both peripheral serum and NP tissue.

## METHODS

Chronic rhinosinusitis with nasal polyps (CRSwNP) was clinically diagnosed according to the established guidelines of the European Position Paper on Rhinosinusitis and Nasal Polyps 2020 (EPOS2020).^16^ We analysed the clinical data of 26 non-allergic CRSwNP patients and 22 controls, who were admitted from December 2020 to November 2021 at The Second Hospital of Shandong University. Inferior turbinate samples from patients without sinus disease undergoing septoplasty or rhinoseptoplasty were collected as controls (controls, n=22). Samples from patients with NPs (non-allergic CRSwNP, n=26) were obtained during functional endoscopic sinus surgery procedures. All samples were divided into two parts: one was placed in formalin for tissue sectioning, and one was frozen in RNA later (Ambion, Austin, TX, USA) for mRNA extraction. All patients underwent serum specific immunoglobulin E (IgE) tests for common inhalant allergens, CT testing, and pulmonary function testing.

### Exclusion criteria

CRSwNP patients with local CRSwNP, posterior nasal polyps, sinus cysts, unilateral lesions, severe systemic diseases, autoimmune diseases, and failure to sign an informed consent form were excluded. Control subjects or patients with CRSwNP who had a history of allergic diseases, such as asthma, allergic rhinitis, and allergic fungal sinusitis, or a positive serum specific IgE test were excluded.

### Ethical Aspect

We briefed all participants in detail on the sample collection process and all participants provided written informed consent following the declaration of Helsinki. All procedures in this study related to human participants followed the ethical standards of the institutional and/or national research committees. The approval of this study was obtained from the institutional review committee of The Second Hospital of Shandong University, Shandong University, China (China approval number: KYLL-2018(KJ)P-0025).

### Hematoxylin and eosin staining (H&E staining) and Immunofluorescence staining (IF staining)

Paraffin blocks were sectioned at 4mm thickness on a microtome (Leica, Wetzlar, Germany), then H&E staining was used to highlight the eosinophils, as well as evaluate epithelial remodeling. Expression of CK5 and BDNF on paraffin sections of nasal biopsies was studied by IF staining. The antibodies for immunofluorescence used were for mouse monoclonal anti-BDNF antibody (ab203573; Abcam) and rabbit monoclonal recombinant anti-CK5 antibody (ab40773; Abcam). The deparaffinized sections were processed with a target retrieval buffer (Dako). The slides were subsequently incubated with primary antibodies at 4°C overnight. They were then incubated with Alexa Fluor 488 or 594 conjugated secondary antibodies (goat anti-mouse or anti-rabbit immunoglobulin G, heavy and light chains; Molecular Probes, Carlsbad, California, USA) at 1:400 in the dark at room temperature for one hour. This was followed by mounting with an anti-fade reagent using 4′, 6-diamidino-2-phenylindole (DAPI) (Molecular Probes). The slides were then analyzed with fluorescent microscopy (Olympus GX51, Tokyo, Japan). The eosinophils, BDNF positive cell count, and CK5 positive cell count were calculated at high power field (HPF) magnification (×400) and 5 fields were randomly selected and analyzed. Then sorted the count of BDNF in descending or ascending order, with the median as the critical point. Groups greater than or equal to the median were considered BDNF^high^, while those below the median were considered BDNF^low^. To have a standardized histologic evaluation of the staining, two researchers independently assessed all cases.

### RNA extraction and Quantitative real-time PCR (qRT-PCR)

Total RNA was extracted from frozen nasal tissues in RNA later (Ambion, Austin, TX, USA). The RNA quantity was measured with a NanoDrop 2000 instrument (Thermo Scientific, Waltham, MA, USA). mRNA levels of CK5, BDNF, and ECP were detected by SYBR Green gene expression assays. The sequences of the CK5, BDNF, ECP, and glycer aldehyde-3-phosphate dehydrogenase (GADPH) primers were as follows: 5′-TGGAAGACTTCAAGAACAAG-3′(forward) and 5′-ATGTAGGCAGCATCTACATC-3′(reverse) for CK5, 5′-CAAAAGTGGAGAACATTT-GC-3′ (forward) and 5′-AACTCCAGTCAATAGGTC-AG-3′(reverse) for BDNF, 5′-AGAGACTGGGAAACATGG-3′(forward) and 5′-GATAATTGTTAATTGCCC-GC-3′(reverse) for ECP and 5′-ACAGTTGCCATGTAGACC-3′(forward) and 5′-TTGAGCACAGGGTACTTTA-3′ (reverse) for GADPH. Relative gene expression was calculated using the comparative 2^–ΔΔCt^ method with the housekeeping gene (GAPDH) as a reference.

### Enzyme-Linked Immunosorbent Assay (ELISA)

To quantify BDNF and ECP protein levels in serum, a commercially available ELISA kit was used according to the manufacturer’s instructions (CUSABIO, Wuhan, China). Plates were read in a microplate reader at 450 nm and 570 nm according to the protocol of the manufacturer, and the concentrations of the samples were determined by comparison with the standard concentration curves.

### Statistical analysis

Statistical analyses were performed with GraphPad Prism v7.0 software (USA, GraphPad Software), and *a p-value* of less than 0.05 was considered statistically significant. To compare the mean of continuous variables between the groups, Mann Whitney test was used to assess significant intergroup variability. Spearman’s rank analysis was used to assess the correlations.

## RESULTS

### Demographics and clinical characteristics

Clinical characteristics were compared between non-allergic NPs (n=26) and controls (n=22). In this study, the median age of those who received endoscopic sinus surgery in NPs was older compared with the control group [50 years (32.23-55.75 years) versus 34 years (18.5 -49 years); *p* < 0.05)]. All other demographic and clinical variables compared between groups were not statistically different.

### More eosinophils in non-allergic NPs tissues

**Fig.1:**
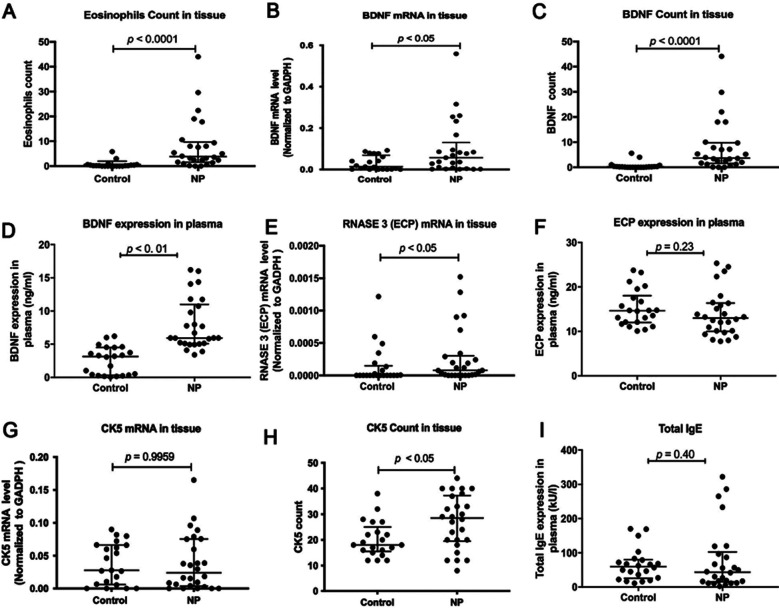
The semi-quantitative expression of paraffin specimens and serum from healthy controls and patients. (A) Eosinophil count in tissues between NPs and controls. (B-D) The mRNA expression, tissue count, and serum expression of BDNF. (E–F) The mRNA and serum expression of RNASE3 (ECP). (G–I) The mRNA expression and tissue count of CK5 (KRT5). (I) Total IgE in serum between NPs and controls.

**Fig.2:**
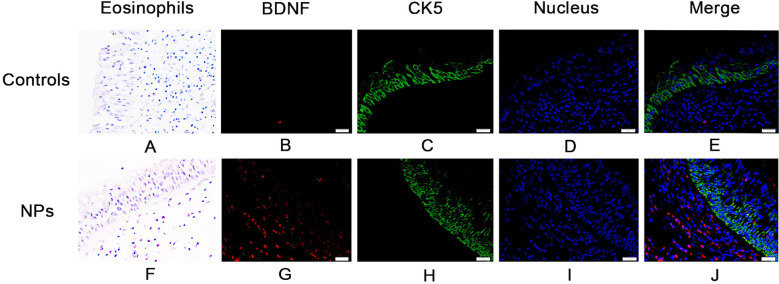
Staining characteristics of healthy controls and patients with NPs. Hematoxylin and eosin staining of eosinophils from healthy controls (A) and patients with NPs (F). BDNF of healthy control subjects (B) and patients with NPs (G) distributed below the epithelium and was stained red. CK5 (KRT5) of healthy control subjects (C) and patients with NPs (H) distributed in the epithelium and was stained green. Nucleus were stained blue with 4,-6-diamidino-2-phenylindole dihydrochloride (D and I). The merge of red BDNF, green CK5 (KRT5), and blue cell nuclei in healthy controls and patients (E and J).

The tissue eosinophil percentage in the NPs was higher than that of the controls (Fig.1A, *p* < 0.001). Eosinophil infiltration by H&E staining was shown in Fig.2A & F. Moreover, an increased percentage of epithelial hyperplasia, goblet cell hyperplasia, and squamous metaplasia were shown in NPs (Table-I).

**Table-I:** Clinical and HE staining characteristics between two groups.

	Control (IT)	Non-allergic NPs	p value
Sample size (No.of patients)	22	26	NA
Age	34	50	<0.05
Median (1^st^-3^rd^ quartile)	(18.5-49)	(32.23-55.75)	
Gender (M/F)	13/9	17/9	NS
Smoking (With/Without)	3/19	6/20	NS
Tissue eosinophilia (percentage) ^‡^	4.5	38.5	<0.001
Epithelial hyperplasia (percentage) ^†^	23.1	57.9	<0.05
Goblet cell hyperplasia(percentage)	9.1	50	<0.01
Squamous metaplasia (percentage)	0	46.7	<0.05

Non-allergic NPs, non-allergic nasal polys, † Epithelium with more than 4 layers was defined as epithelial hyperplasia, ‡ The count of eosinophils exceeding 10 was categorized as eosinophilia, Differences in categorical variables between the two groups were compared using chi-square test or Fisher’s exact test, the difference of age was compared with independent t-test, P <0.05 was considered statistically significant unless otherwise stated; NA, not applicable; NS, no significant differences.

### Elevated BDNF expression in both tissue and serum of non-allergic NPs patients

The relative expression of BDNF mRNA in tissue was significantly higher in non-allergic NPs (Fig.1B, *p* < 0.05). BDNF staining by immunohistochemistry was shown in Fig.2 B & G. The localization of mature BDNF^+^ cells was mainly in the sub-epithelial layer. Significantly higher BDNF protein level in tissue was found in non-allergic NPs (Fig.1C, *p* < 0.0001). BDNF levels were also found to be increased in serum in non-allergic NPs than in controls (Fig.1D, *p* < 0.01).

### ECP and CK5 expression between two groups

The ECP mRNA expression in tissue was significantly higher in non-allergic NPs than in control (Fig.1E, *p* < 0.05), but there was no statistical difference regarding CK5 mRNA levels (Fig.1G, p = 0.9959). The localization of CK5 protein level in tissue was mainly in the epithelium (Fig.2 C & H), which was higher in non-allergic NPs (Fig.1H, *p* < 0.05). However, regarding ECP and total IgE in serum, we could not find a statistical difference between the two groups. (Fig.1 F & I, *p =* 0.23, *p =* 0.40).

### Elevated eosinophils and ECP mRNA expression in BDNF^high^ subgroups in NPs

**Fig.3:**
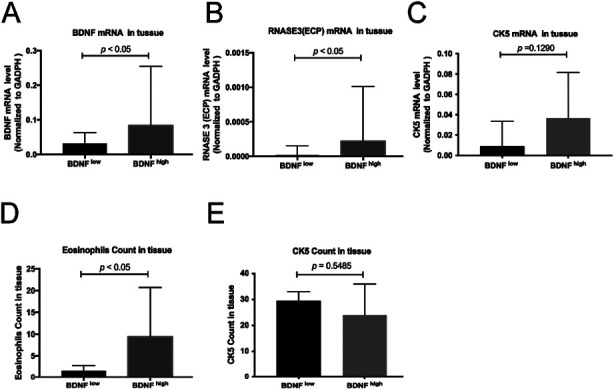
The semi-quantitative expression in the subgroup (BDNF^low^ and BDNF^high^). (A–C) The mRNA expressions of BDNF, RNASE3(ECP) and CK5 (KRT5) between the BDNF^low^ and BDNF^high^. (D–E) The count for eosinophils and CK5 (KRT5).

We divided the non-allergic NPs patients into BDNF^high^ and BDNF^low^ subgroups by median count in tissue. We found that the eosinophils infiltration, ECP mRNA expression level, as well as BDNF mRNA level, were significantly increased in the BDNF^high^ subgroup (Fig.3A, B & D, *p* < 0.05, *p* < 0.05, *p* < 0.05). However, no difference in CK5 protein and mRNA level was found (Fig.3 C & E).

### Clinical characteristics in BDNF^low^ and BDNF^high^ subgroups in non-allergic NPs

**Fig.4:**
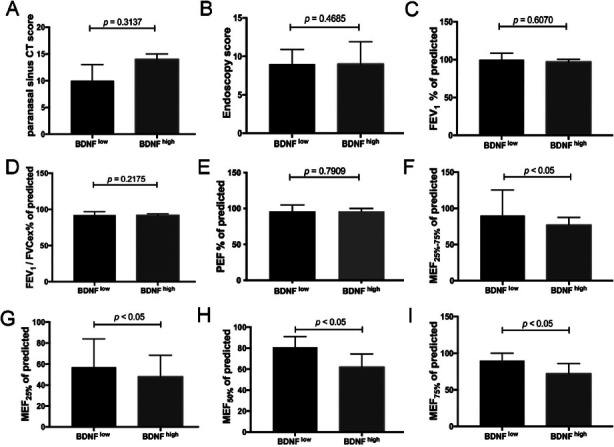
The clinical features in the subgroup (BDNF^low^ and BDNF^high^). (A) The Lund-Mackay CT scores between the BDNF^low^ and BDNF^high^. (B) The endoscopy scores between the BDNF^low^ and BDNF^high^. (C–I) The related indicators of lung function (FEV_1_% of predicted, FEV_1_/FVC_ex_% of predicted, PEF% of predicted, MEF_25%-75%_, MEF_25%_, MEF_50%_, and MEF_75%_) in BDNF^high^ and BDNF^low^ subgroups.

We found a significant decrease of MEF_25-75%_ of predicted, MEF_25%_ of predicted, MEF_50%_ of predicted, and MEF_75%_ of predicted in BDNF^high^ subgroup compared with BDNF^low^ subgroup (Fig.4 F, G, H & I, *p* < 0.05, *p* < 0.05, *p* < 0.05 and *p* < 0.05). No significant differences regarding Lund-Mackay CT scores, endoscopy scores, FEV_1_% of predicted, FEV_1_/FVCex% of predicted, and PEF% of predicted (Fig.4 A, B, C, D & E) were found between the subgroups.

### Correlation analysis of BDNF and clinical characteristics in non-allergic NPs

**Fig.5:**
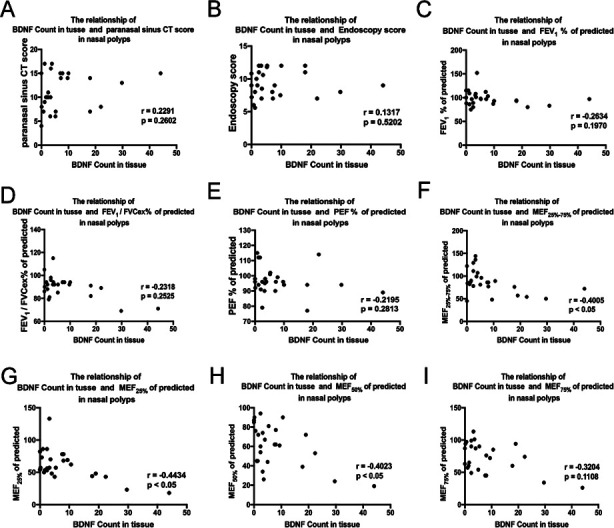
The correlations between BDNF count and relative clinical factors. The correlations between BDNF count and the Lund-Mackay CT scores (A), the endoscopy scores (B), and the related indicators of lung function (FEV_1_%, FEV_1_/FVC_EX_%, PEF%, MEF_25%-75%_, MEF_25%_, MEF_50%_, and MEF_75%_) (C-I).

Significantly negative correlations between BDNF count in tissue and MEF_25-75%_ of predicted, MEF_25%_ of predicted, MEF_50%_ of predicted were found in non-allergic NPs (Fig.5 F, G & H, *p* < 0.05, *r* = -0.4005; *p* < 0.05, *r* = -0.4434; *p* < 0.05, *r* = -0.4023). No correlations between BDNF count in tissue and Lund-Mackay CT scores, endoscopy scores, FEV_1_% of predicted, FEV_1_/FVC_ex_% of predicted, PEF% of predicted, and MEF_75%_ of predicted were found (Fig.5 A, B, C, D, E & I).

## DISCUSSION

In this study, we compared the expression of BDNF, CK5, and ECP in non-allergic NPs patients and healthy controls. The results demonstrated that elevated BDNF was expressed in both peripheral blood and nasal mucosa in non-allergic NPs. This study also gave evidence for elevated eosinophils infiltration and ECP mRNA expression in the BDNF^high^ subgroup compared with the BDNF^low^ subgroup. These results suggested that BDNF may be involved in NPs’ inflammation, especially in eosinophil infiltration NPs.

BDNF is a secretory protein that belongs to the neurotrophin family. Some researchers reviewed that BDNF was associated with Alzheimer’s disease, Parkinson’s disease, Huntington’s disease, amyotrophic lateral sclerosis, major depressive disorder, schizophrenia, epilepsy, bipolar disorder, glioblastoma, and intracranial glioma.^17^-^19^ Pathological examinations have shown that there is an excessive expression of BDNF in some types of human cancer, particularly in the prostate, lung, breast, and surrounding tissues.^20^ Some researchers found that exercise, morphine, and the concurrent effect of morphine and exercise had a significant positive effect on BDNF.^21^ In addition to playing an important role in neural processes, BDNF may be a potential key determinant in allergic diseases and NPs patients. Increased serum levels of BDNF have been found in patients with allergic rhinitis, atopic dermatitis, and allergic asthma compared with controls.^22^-^24^ In the animal model, BDNF was proven to contribute to airway hyperreactivity and allergic inflammation.^25^ Jin has previously shown that functional BDNF gene variants increased the risk of moderate-to-severe allergic rhinitis.^26^ Katherina showed that polyp epithelial cells expressed higher BDNF levels compared to controls, and BDNF was a peripheral blood biomarker of asthma with aspirin sensitivity.^23^ Charles pointed out that mean BDNF concentration was decreased in CRS, especially in patients with polyps.^27^ Therefore, being a part of the neurotrophic family, BDNF has the ability to regulate the body’s immune response and is associated with allergic diseases and NPs process. However, we can not prove whether the effect of BDNF on NPs is disrupted by allergic diseases in this case. In our study, NPs patients were enrolled in pure form (serum specific immunoglobulin E testing was performed to exclude allergic patients) to analyze the contribution of BDNF in non-allergic NPs. The localization of mature BDNF^+^ cells in the sub-epithelial layer was demonstrated. Higher BDNF protein and mRNA expression were found in non-allergic NPs tissues compared within controls, as well as elevated serum BDNF protein level, which suggested that BDNF may have certain physiological functions in non-allergic NPs patients, and the effects of BDNF on the local site may join the systemic circulation.

Next, we analyzed the eosinophil infiltration, ECP expression, and CK5 expression in non-allergic NPs. ECP is an eosinophil-derived substance contained in granules, which can be released during inflammation and can cause various biological effects. It is worth noting that eosinophils can not only secrete neurotrophins (BDNF), but also secrete chemokines and cytokines, such as ECP. In this experiment, more eosinophils and elevated ECP mRNA levels were found in non-allergic NPs, but there was no statistical difference between NPs and controls regarding serum ECP. However, high serum ECP concentrations in patients with chronic rhinosinusitis without allergic rhinitis were found in Kim’s study.^28^ Studies have revealed that the levels of ECP in nasal secretions are considerably higher than those in the serum.^29^ We speculated that ECP may show its effect on the local site without joining the circulation, so priority should be given to determining ECP levels in nasal secretions over those in the serum. The possibility of taking serum samples in the non-allergic season could also affect the results of our study. Moreover, eosinophils and ECP mRNA expression levels in BDNF^high^ subgroup were significantly increased compared with BDNF^low^ subgroup. These imply that BDNF, ECP, and eosinophils could work together to affect the pathogenesis of non-allergic NPs. In addition, we all know that CK5 plays an extremely important role in NPs, which is mainly expressed in the basal cells.^30^ But so far, the role of CK5 in non-allergic NPs has not been reported. In our study, the higher CK5 count was found in non-allergic NPs, but there was no statistical difference regarding CK5 mRNA level. No difference in CK5 protein and mRNA level was found between BDNF^high^ and BDNF^low^ subgroups. These can be attributed to that this study was carried out in a solitary institution with a limited number of patients, which might bring about selection bias and lack the ability for external validation.

Some experts have found that CRSwNP patients with tissue eosinophilia presented with higher Lund-Mackay scores and endoscopy scores.^31^ Rahim demonstrated that NPs patients with allergic disease had higher Lund-Mackay scores.^32^ However, in the present study, we did not find obvious differences in Lund-Mackay CT scores and endoscopy scores between the BDNF^high^ subgroup and the BDNF^low^ subgroup in non-allergic NPs. It might be conducted with the small sample size of this study. FEV_1_% plays the key role that reflected the maximum ventilation function, MEF_25-75%_, MEF_25%_, MEF_50%_, and MEF_75%_ could exactly reflect the situation of airway obstruction, especially small airway abnormality.^33^ Clinical characteristics between BDNF^high^ subgroup and the BDNF^low^ subgroup were analyzed in this study. We found a significant decrease of MEF_25-75%_, MEF_25%_, MEF_50%_, and MEF_75%_ in the BDNF^high^ subgroup compared to the BDNF^low^ subgroup. Negative correlations between BDNF count in tissue and MEF_25-75%_, MEF_25%_, and MEF_50%_ were found. This suggested BDNF might cause impairments of small airway ventilation function, which was mainly related to the existence of NPs. Previous studies have reported that intervening against BDNF could help reduce inflammation and restore the balance between oxidation and antioxidation in the airway.^34^ When it comes to the underlying mechanism, an elevation in BDNF could potentially enhance airway irritability through the nervous system, adjust bronchodilator responses derived from the epithelium, or heighten airway smooth muscle [Ca2+]_i_ and contractility, particularly in the presence of inflammatory cytokines.^35^

### Limitations

First of all, the sample size is relatively small, with only 22 control groups and 26 experimental groups. Consequently, larger sample sizes are required to obtain more statistically robust results. Second, we take serum samples in the non-allergic season, which may affect the results. Considering the dynamic nature of allergies, it would be beneficial to collect samples in allergic seasons.

## CONCLUSION

We have shown that the increased expression of BDNF in non-allergic NPs was associated with tissue eosinophilia. BDNF may have both local and systematic effects on non-allergic NPs pathogenesis. Thus, BDNF may be a possible therapeutic target or an indicator for the management of NPs, especially eosinophilic NPs.

### Authors Contribution:

**XZ:** Conception, stud design, coordination of project, finalizing the manuscript.

**LS:** Conception, literature review, conception and study design, and also accountable for the accuracy and integrity of the work.

**YW:** Data collection and analysis, preparing the manuscript.

**QZ:** Data review, literature search.

All authors read, revised, and approved the final manuscript.
